# Exploring methodological frontiers in laminar fMRI

**DOI:** 10.1093/psyrad/kkae027

**Published:** 2024-11-22

**Authors:** Yuhui Chai, Ru-Yuan Zhang

**Affiliations:** Beckman Institute for Advanced Science and Technology, University of Illinois at Urbana-Champaign, Urbana 61801, Illinois, USA; Brain Health Institute, National Center for Mental Disorders, Shanghai Mental Health Center, Shanghai Jiao Tong University School of Medicine and School of Psychology, Shanghai 200030, the People Republic of China

**Keywords:** Laminar fMRI, cortical layer, cortical depth, high-resolution fMRI

## Abstract

This review examines the methodological challenges and advancements in laminar functional magnetic resonance imaging (fMRI). With the advent of ultra-high-field MRI scanners, laminar fMRI has become pivotal in elucidating the intricate micro-architectures and functionalities of the human brain at a mesoscopic scale. Despite its profound potential, laminar fMRI faces significant challenges such as signal loss at high spatial resolution, limited specificity to laminar signatures, complex layer-specific analysis, the necessity for precise anatomical alignment, and prolonged acquisition times. This review discusses current methodologies, highlights typical challenges in laminar fMRI research, introduces innovative sequence and analysis methods, and outlines potential solutions for overcoming existing technical barriers. It aims to provide a technical overview of the field's current state, emphasizing both the impact of existing hurdles and the advancements that shape future prospects.

## Introduction

Since its development in the early 1990s by pioneers such as Bandettini *et al*. (1992); Belliveau *et al*. (1991); Kwong *et al*. (1992); and Ogawa *et al*. (1992, 1990), functional magnetic resonance imaging (fMRI) has transformed into an essential tool in human cognitive neuroscience research. The technique enables non-invasive studies of brain function and has seen significant advancements over the past decade with the introduction of ultra-high-field (≥7 T) human MRI scanners. These advancements have dramatically improved fMRI spatial resolution into the sub-millimeter domain (Koopmans *et al*., 2011; Polimeni *et al*., 2010; Sanchez-Panchuelo *et al*., 2010), enhancing our ability to differentiate fMRI signals across cortical depth and providing a more granular understanding of brain function at the level of cortical layers (Feinberg *et al*., 2018; Huber *et al*., 2017a; Muckli *et al*., 2015; Weldon and Olman, 2021).

Although the cortical depth delineated in high resolution fMRI does not directly correspond to each cytoarchitectonically defined cortical layer, it provides highly realistic cortical contours along laminar directions (Waehnert *et al*., 2014). These cortical layers are recognized for their distinct functional roles (Lamme *et al*., 1998; Self *et al*., 2017): feed-forward, bottom-up signals primarily project to the middle granular layer, while feedback, top-down signals are predominantly found in superficial and/or deep layers (Felleman and Van Essen, 1991; Norris and Polimeni, 2019; Self *et al*., 2017). Such distinctions allow for the exploration of both layer-specific activity (Chai *et al*., 2021b; Finn *et al*., 2021, 2019; Lawrence *et al*., 2017) and connectivity (Chai *et al*., 2024b; Huber *et al*., 2021a; Rockland, 2017) using high resolution fMRI, aiding in the differentiation of bottom-up and top-down cognitive processes and elucidating the functional hierarchy across cortical areas (Stephan *et al*., 2017).

Moreover, laminar fMRI facilitates bridging the macroscopic observations typical of conventional fMRI and behavior studies with the microscopic insights gained from animal and *ex vivo* research (e.g. extracellular recordings and microscopic imaging) (Self *et al*., 2017; Yang *et al*., 2021). By exploring brain function at this mesoscopic scale, laminar fMRI not only deepens our understanding of cortical micro-circuitry in both health and disease (Huber *et al*., 2023; Stephan *et al*., 2017) but also integrates these insights into broader human cognitive neuroscience investigations.

## Technical challenges in laminar fMRI

Despite its significant potential for cognitive neuroscience, laminar fMRI faces several technical challenges that hinder achieving adequate sensitivity, specificity, and accuracy at this mesoscopic scale. These challenges, summarized in Table 1 and elaborated on next, range from issues common in high-field MRI to those particular to laminar fMRI.

1. Poor laminar specificity: Gradient echo (GE) blood-oxygen-level-dependent (BOLD) contrast is commonly used for human functional brain mapping (Ogawa *et al*., 1990) due to its high sensitivity. However, GE-BOLD signal is prone to draining vein effects, where signals from ascending and pial veins dominate, masking the laminar-specific microvasculature signals (Kim and Ogawa, 2012). This results in a disproportionate signal weighting across layers, with superficial layers being most heavily affected (Uludağ and Blinder, 2018). Consequently, interpretating individual layer activity with GE-BOLD becomes challenging, complicating the clarity of laminar fMRI results (Kay *et al*., 2019).
2. Low sensitivity: When transitioning from traditional fMRI with an example resolution of 3 mm isotropic to laminar fMRI at 0.75 mm isotropic, there is an inherent drop in voxel signal intensity by a factor of 64, significantly reducing sensitivity. Furthermore, due to the poor laminar specificity in BOLD, laminar fMRI research is increasingly exploring cerebral blood volume (CBV) and cerebral blood flow (CBF) based measurements, which leads to a further reduction in sensitivity. CBV-based fMRI methods such as VASO inherently exhibit low signal to noise ratio (SNR), with only 10–20% of tissue signal remaining at the time of blood nulling (Hua *et al*., 2009), leading to sensitivity levels typically <50% of those seen with BOLD. CBF-based arterial spin labeling (ASL) methods show even lower sensitivity, typically ranging from 10 to 20% of BOLD levels (Huber *et al*., 2017b).
3. Low spatial accuracy: Because the contrast between gray matter (GM) and white matter (WM) is relatively poor in echo planar imaging (EPI), these boundaries are often defined in anatomical volumes acquired with different pulse sequences (usually MP2RAGE: Marques *et al*., 2010) each with unique geometric distortions. When anatomical images are acquired separately from the functional data, researchers face the additional challenge of optimizing registration between different datasets each having unique geometric distortions. This discrepancy can lead to mismatches in the definition of cortical depths/layers in anatomical and functional images (Weldon and Olman, 2021).
4. Limited coverage: To date, most layer fMRI studies have been confined to local brain areas using a task design, largely due to the methodological limitation of layer fMRI sequence. Although non-BOLD fMRI contrasts, such as CBV and CBF based measurement (Chai *et al*., 2024b, 2020; Huber *et al*., 2021a, 2017b), have been shown to be more layer specific, their acquisition rate is more than twice slower than BOLD, which limits the slice number within an acceptable repetition time for fMRI.
5. Long repetition time (TR): As spatial resolution and coverage increase in laminar fMRI, there is a corresponding extension in TR. In BOLD-fMRI, the minimal TR is largely determined by the limits of gradient performance and the extent of achievable acceleration via parallel imaging techniques. Non-BOLD fMRI modalities, such as those based on CBV and CBF, necessitate additional acquisition time, due to the slower generation of intravascular contrast and the need for interleaved acquisitions to correct for BOLD effects (Huber *et al*., 2021a).
6. Lack of standardized tools: The adoption of layer fMRI techniques is further complicated by the absence of standardized analysis tools, leading researchers to relay on a combination of many different toolboxes, such as LAYNII (Huber *et al*., 2021b), ANTs (Avants *et al*., 2009), AFNI/SUMA (Cox, 1996), FreeSurfer (Fischl, 2012), SPM (Penny *et al*., 2011), FSL (Jenkinson *et al*., 2012), and various home-made programs, often necessitating extensive manual corrections.
7. Common high-field MRI issues: In high-field MRI, issues such as specific absorption rate (SAR) constraints, B0 and B1 + inhomogeneity present significant challenges. B0 inhomogeneity can lead to signal loss and geometric distortions during fMRI EPI acquisitions (Stockmann and Wald, 2018). Additionally, B1 + inhomogeneity and SAR constraints affect the performance of radio frequency (RF) pulses, especially refocusing pulses in spin-echo and preparation pulses in CBV- and CBF-based fMRI (Huber *et al*., 2018), affecting both the contrast and measurement reliability.

**Table 1:** Challenges to overcome in laminar fMRI.

Challenges	Potential solutions
Poor specificity of BOLD fMRI	Utilization of CBV or CBF based measurement, BOLD correction for drain vein effects
Reduced signal intensity at higher resolutions (e.g. 64× reduction from 3 to 0.75 mm isotropic)	Use of high-field systems (≥7 T), 3D-EPI for better sensitivity than 2D-EPI at mesoscale
Poor spatial accuracy of layer/depth definition due to registration issue at mesoscale	Conducting anatomical imaging and all analysis in native fMRI/EPI space to avoid anatomical-functional registration
Coverage limitations in current layer-fMRI sequence	Dynamic blood nulling for CBV or CBF imaging, high imaging acceleration with EPI segmentation
Large image matrices (e.g. 320 × 320 × 200 for whole-brain 0.5 mm fMRI) and long repetition times	Advanced imaging acceleration, such as skipped-CAIPI, deep learning reconstruction, spatiotemporal (*k,t*)-space acceleration
Lack of standardized analysis tool	Reliance on a combination of toolboxes such as LAYNII, ANTs, ITK-SNAP, AFNI/SUMA, FreeSurfer, SPM, FSL, and custom scripts, often requiring extensive manual corrections
Common high-field MRI issues (B1 and B0 field inhomogeneity, SAR)	Applications of advanced B0 and B1 shimming, adiabatic RF pulses

## Sequence methods for layer-specific functional measurement

To address the issue of laminar specificity, numerous sequence development efforts have been undertaken, aiming to either refine BOLD signals with less macrovascular bias or employ non-BOLD contrasts. In the following is a brief overview of contrast weightings across several example layer fMRI sequences, with their respective advantages and limitations summarized in Table 2.

### BOLD

As introduced before, the GE-BOLD signal is susceptible to the draining vein effect, which significantly dilutes its layer-specific microvasculature signals with dominant signals from ascending and pial veins (Kim and Ogawa, 2012). However, because of its ease of implementation and high sensitivity, it remains as the workhorse for fMRI studies that seek the sub-millimeter resolution necessary to resolve layer-dependent fMRI signals. The contaminations from draining and pial veins in laminar BOLD signals might be modelled as much as possible (Heinzle *et al*., 2016; Uludag and Havlicek, 2021) and hence reduced in post-processing (Huber *et al*., 2021b).

To reduce the macrovascular contribution to BOLD signal in acquisition, one solution is to employ spin-echo instead of gradient echo sequences. Hemoglobin within blood vessels causes microscopic magnetic field distortions, leading to spin dephasing in proportion to the blood oxygenation level. In spin-echo BOLD (SE-BOLD), spins out of phase due to magnetic field distortions caused by large veins can be refocused by the 180° RF pulses, except those near capillaries that diffuse to slightly different magnetic field within the echo time. As a result, SE-BOLD effectively eliminates large vein extra-vascular BOLD signal (Yacoub *et al*., 2007), thereby providing superior laminar specificity over T_2_*-weighted techniques like GE-BOLD. In practice, however, the T_2_ -weighted signal from SE-BOLD is typically sampled with a gradient echo EPI readout, imposing additional T_2_*-weighting. The length of the EPI acquisition window determines the relative prominence of T_2_* contrast: longer acquisition windows increase the relative contribution of T_2_*, while shorter acquisition windows highlight the apparent T_2_-influence. Techniques such as SE multi-shot EPI (Goense and Logothetis, 2006) and T_2_-prepared sequences with short gradient echo readouts (Pfaffenrot *et al*., 2021) have been employed to minimize T_2_* weighting relative to T_2_ effects.

**Table 2:** Pros and cons of different laminar fMRI sequence methods.

Methods	Pros	Cons
GE-BOLD	High sensitivity, easy to implement, short TR	Poor laminar specificity due to draining and pial veins
SE-BOLD	SE-EPI: reduced macrovascular bias; T2-prep: signal primarily from small vessels	SE-EPI: T2* effects vary with EPI acquisition length; T2-prep: low sensitivity
VASO	High laminar specificity, excellent tissue contrast facilitating layer analysis	Sensitivity ≤50% of BOLD, TR more than twice as long as GE-BOLD
ASL	Best laminar specificity among listed methods, provides quantitative measurements	Sensitivity 10–20% of BOLD, TR longest among listed methods
VAPER	High laminar specificity, dynamic pseudo-continuous blood nulling throughout acquisition thus not sensitive to inflow effects	Sensitivity ≤50% of BOLD, TR more than twice as long as GE-BOLD

### CBV and CBF based fMRI

Selective sensitivity of CBV and CBF has been demonstrated to provide improved spatial specificity to the activated parenchyma and better localization of layer-specific activity in animal models (Jin and Kim, 2008). Thus, CBV- and CBF-based fMRI measurements, such as VASO (vascular space occupancy) (Huber *et al*., 2014; Lu *et al*., 2005), ASL (Kashyap *et al*., 2021; Shao *et al*., 2021), and VAPER (integrated blood volume and perfusion) (Chai *et al*., 2020) imaging, have been explored or developed, offering more layer-specific information than traditional BOLD imaging.

**Figure 1:**
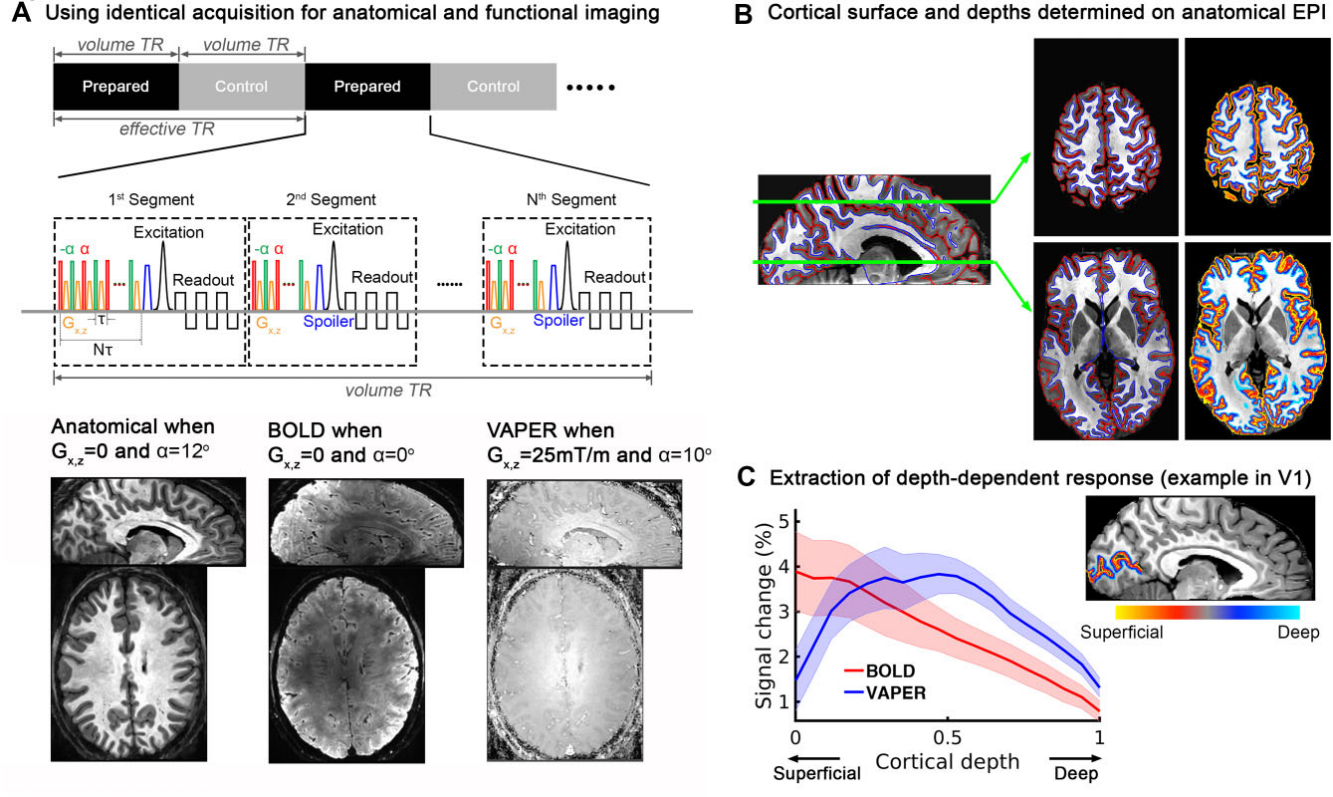
One example of layer fMRI data acquisition strategies. (**A**) Anatomical MT-weighted, functional BOLD and VAPER images acquired using an identical EPI acquisition design, which all images matched during acquisition. (**B**) Determination of cortical surface and depths in the native fMRI space based on anatomical MT-EPI images. (**C**) Extraction of laminar profile of fMRI response. Adapted from Chai *et al*. (2024b).

VASO: VASO is currently one of the most used non-BOLD sequences for layer-specific fMRI. This technique takes advantage of the difference in longitudinal relaxation times (T_1_) between tissue and blood to measure the remaining extravascular water signal at the zero-crossing point along blood T_1_ recovery following an inversion pulse. Unlike fMRI sequences that depend on susceptibility contrasts such as T_2_* weighting, VASO utilizes *T*_1_ rate differences, thereby providing excellent tissue contrast, which greatly enhances layer-specific fMRI data analysis. This technique has been successfully implemented to map laminar activity and connectivity across various brain areas (Finn *et al*., 2021, 2019; Huber *et al*., 2017a) and at the whole-brain level (Huber *et al*., 2021a) in studies conducted at both 7 T ( Huber *et al*., 2017a ) and 9.4 T (Huber *et al*., 2018).
ASL: ASL magnetically labels arterial blood water that perfuses into the local tissue (Williams *et al*., 1992), and it is the only non-invasive method that allows functional CBF measurement in humans . Perfusion contrast by ASL offers even better specificity than VASO. The ASL signal is localized close to the site of neural activation as most of the labeled arterial water exchanges with tissue water in capillaries. Another advantage, compared to BOLD and VASO fMRI, is that ASL perfusion contrast offers the unique capability for quantitative CBF measurements both at rest and during task activation (Borogovac and Asllani, 2012). However, the major limitation of ASL perfusion is its low sensitivity, complicating its use in layer fMRI applications (Huber *et al*., 2017b). Although there have been a few laminar fMRI studies using ASL in humans at 7T (Kashyap *et al*., 2021; Shao *et al*., 2021), it is still challenging to apply it in a more widespread manner for layer fMRI due to its low sensitivity.
VAPER: To take advantage of the good specificity of both CBV and CBF for layer-dependent fMRI research, we have proposed using DANTE (Delay Alternating with Nutation for Tailored Excitation) pulse trains (Li *et al*., 2012) combined with 3D-EPI imaging technique (Poser *et al*., 2010; Stirnberg and Stöcker, 2020) to acquire an integrated VASO and perfusion contrast (VAPER) (Chai *et al*., 2020). The DANTE pulse train serves dual purposes: nulling blood to provide a VASO contrast, and labelling blood for perfusion weighted imaging as in the ASL. The sequence acquires fMRI images between two states (Fig. 1): during and after DANTE blood suppression. During DANTE, blood signals in the microvasculature of the human brain are nearly nulled to achieve a VASO contrast. After DANTE, fresh blood from outside of the coil coverage flows into the image microvasculature and replaces the nulled blood, generating a perfusion contrast. The signal difference between these two states generate an integrated VASO and perfusion contrast (VAPER). This innovative approach has been demonstrated by successful measurements of layer-specific fMRI activity across primary sensory areas such as visual (Chai *et al*., 2024a), motor (Chai *et al*., 2020), and auditory (Chai *et al*., 2021b) cortices, and connectivity at the whole-brain level (Chai *et al*., 2024b).

## The essential role of anatomical reference imaging in native fMRI space

Layer-specific fMRI not only requires the acquisition of layer-specific functional data but also depends critically on precise anatomical reference imaging. This necessity stems from the need to delineate the inner (gray matter–white matter interface) and outer (gray matter–cerebrospinal fluid interface) boundaries of the cerebral cortex and to assign measured functional data to specific cortical depths with exceptional accuracy. Most fMRI sequences, except for VASO, do not provide sufficient contrast between tissue types, making it difficult to define gray matter boundaries and cortical depths directly from functional images alone. Traditionally, the definition of cortical layers or depths has relied on anatomical reference images obtained with different acquisition techniques, leading to discrepancies due to geometric distortions (Polimeni *et al*., 2017; Weldon and Olman, 2021).

Even though the concept of distortion-matched anatomical imaging for fMRI analysis in native EPI space has been explored in multiple studies previously (Brammer *et al*., 1997; Chai *et al*., 2021a; Grabner *et al*., 2014; Kashyap *et al*., 2017; Renvall *et al*., 2016; Sanchez Panchuelo *et al*., 2021; van der Zwaag *et al*., 2018), most layer fMRI studies still predominantly rely on an anatomical images that differ in contrast or acquisition from functional images. This mismatch necessitates co-registration between functional and anatomical datasets, which blurs functional data and is imprecise at the cortical layer level.

To overcome these challenges, it is preferable to acquire both the anatomical and functional data using the same acquisition design. A method such as a T1-weighted EPI image with inversion recovery (IR) preparation has been suggested, as it matches the distortion characteristics of the functional EPI data (Huber *et al*., 2017a ; Kashyap *et al*., 2017; Renvall *et al*., 2016). To ensure optimization of inversion time, this method has limited acquisition window and brain coverage when preceding a volume acquisition with a single inversion pulse. In high-resolution fMRI, the acquisition time for a single 3D volume image is relatively long. To improve the available sampling time, 3D-EPI volume acquisition can be divided into multiple blocks and each of them is prepared with an IR module as in T1-2-3D-EPI (van der Zwaag *et al*., 2018), or 2D-EPI slices can be reordered differently after each IR as in multiple inversion-recovery time EPI (MI-EPI, 2D) (Renvall *et al*., 2016).

Alternatively, integrating magnetization transfer (MT) weighted imaging with the fMRI acquisition techniques can generate high-quality anatomical images in native fMRI space. In the human brain, a relatively large fraction of macromolecular hydrogen protons (MP) (*f* ∼0.2–0.3) is found in white matter (WM), while this number is smaller in gray matter (GM) (*f* ∼0.1) (van Gelderen *et al*., 2017). Through magnetization transfer (MT) with water hydrogen protons (WP), MPs can dramatically affect the MRI signal and thus different MP fractions in GM and WM will lead to different MRI signal intensities. We have developed a MT weighted anatomical EPI technique, with the EPI design identical to the functional acquisition (Chai *et al*., 2021a, 2021b). This approach ensures that the anatomical and functional images are naturally matched during acquisition, providing sufficient gray-white matter contrast to perform all analysis in the native fMRI space without the need for distortion correction and anatomical-functional co-registration, thereby achieving the highest spatial accuracy in analysis (Fig. 1).

## Analysis strategies to enhance laminar specificity and sensitivity

A general principle in layer fMRI analysis, which distinctly sets it apart from traditional resolution fMRI, is the dual objective to minimize data blurring across cortical depth while maximizing sensitivity. These goals can sometimes be contradictory. For instance, image smoothing can enhance sensitivity but at the cost of blurring the data, thereby reducing laminar specificity. Here, we review several layer-specific fMRI analysis strategies designed to balance these objectives.

### Upsampling data in volume and surface space

Upsampling data in volume and surface space is an effective strategy to mitigate spatial blurring that typically occurs when interpolating between processing stages. This approach is essential for maintaining spatial specificity and is routinely applied to critical layer analysis steps such as determining cortical depths and projecting volumetric data onto cortical depth surfaces. For instance, when calculating cortical depth in volume space, spatial upsampling of the fMRI data is advantageous to define laminar depths on a more refined grid than that with the original EPI resolution, which could avoid singularities at the edges in angular voxel space and resolve the laminar signature with a better specificity. Similarly, for surface-based analysis, such as in our prior layer-specific connectivity study (Chai *et al*., 2024b), we have upsampled the functional volumetric data by a factor of 5 before projection and increased the vertex density (refinement iteration of 1) of each cortical depth surface to minimize resolution loss during the projection between volume and surface space. Moreover, volume upsampling has also proven to improve motion estimation accuracy and reduce data blurring in preprocessing step of motion correction (Wang *et al*., 2022).

### Cortical-depth specific smoothing at individual level

Given the low temporal SNR in high-resolution layer fMRI data, cortical-specific smoothing has emerged as a beneficial strategy to enhance signal sensitivity while preserving spatial information across cortical depths. This layer-specific smoothing employs a Gaussian kernel, with signal leakage strictly confined within the same cortical depth, preventing cross-layer contamination among proximate voxels in Euclidean space (Huber *et al*., 2017a; Polimeni *et al*., 2017). This smoothing strategy has been implemented in several toolboxes, such as LayNii's LN_LAYER_SMOOTH (Huber *et al*., 2021b) by restricting the smoothing kernel across voxels whose centroids are located in the same cortical depth, and AFNI's SurfSmooth by applying smoothing within the surface of each cortical depth.

### Cortical-depth specific surface registration for group layer fMRI analysis

Due to the low sensitivity at high resolution, repeated averaging across individuals is necessary to attain functional statistical significance. However, due to the variable curvature of the cortical ribbon among individuals (Zilles *et al*., 1997), it is very challenging to spatially align and averaging high-resolution fMRI data across subjects for group-level layer-specific analysis. Traditional volume registration methods tend to blur information across cortical depths. We have proposed an innovative cortical depth-specific surface registration method, which maintains signal laminar specificity by aligning cortical surface of each cortical depth to a group-averaged surface of each cortical depth, thus preserving the unique laminar signatures during group analysis. This analysis strategy has enabled the construction of group-level brain map of fMRI connectivity layer patterns, enhancing our understanding of the complex architecture of brain networks (Chai *et al*., 2024b).

### Using columnar analysis to better define laminar analysis ROI

**Figure 2:**
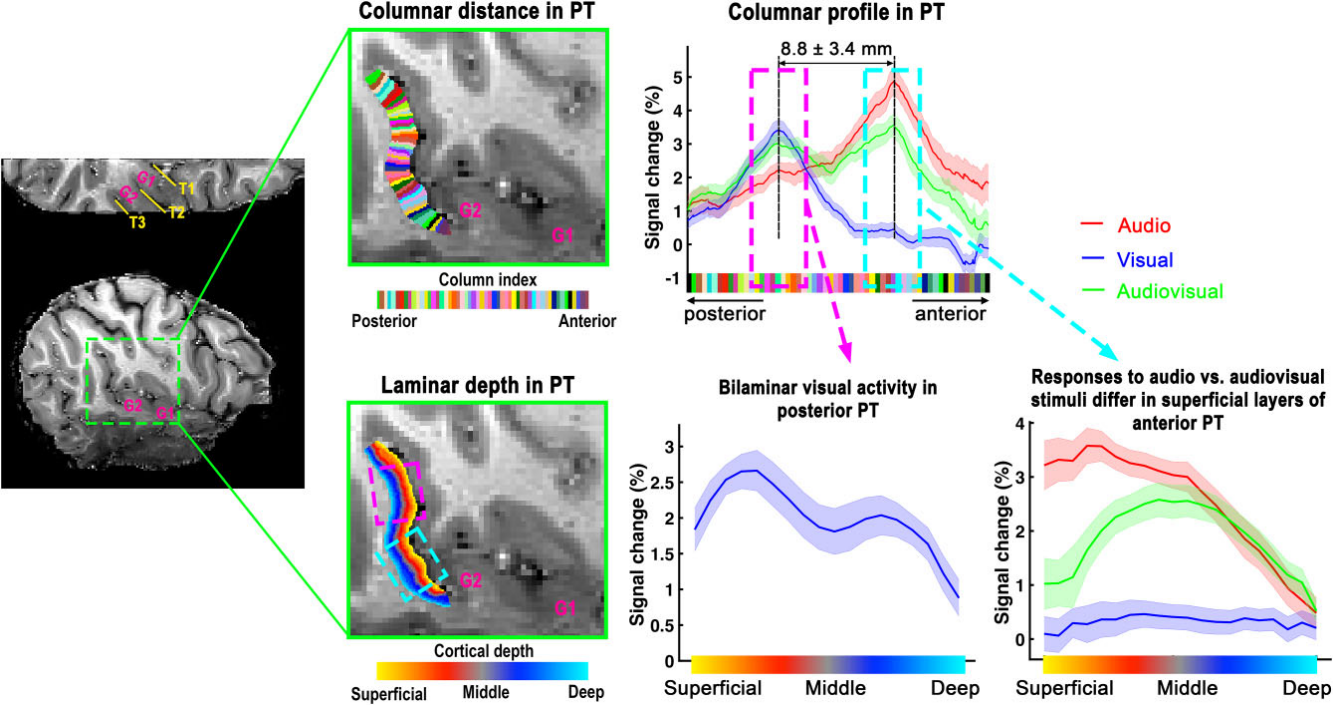
Columnar and laminar distribution of sensory-specific influence in human PT. Left: underlays show the anatomical EPI images that were obtained using an identical acquisition with functional images. Based on G1/G2 (first and second transverse gyrus) landmarks, auditory cortex can be divided into three sub-fields: T1, T2, and T3 (from anterior to posterior). PT is located in T3. Top: columnar distance in PT is assessed, showing response variations across columns due to differing sensory inputs. This columnar profile enables the delineation of subfields within PT, as anterior (marked by light blue dashed rectangle) vs. posterior (marked by magenta dashed rectangle) regions. Bottom: distinct laminar-specific response in anterior vs. posterior PT. Adapted from Chai *et al*. (2021b).

While typical layer fMRI focuses on grouping voxels by relative cortical depth to the gray matter surface, columnar analysis considers voxels in the orthogonal direction to cortical layers. In brain regions characterized by distinct topographical distributions, columnar response profiles can be used to identify locations for layer-specific responses, as laminar profiles can vary significantly across different subfields (Huber *et al*., 2020; Persichetti *et al*., 2020). For instance, in our analysis of multisensory influences in the human planum temporale (PT) (Chai *et al*., 2021b), we observed that different sensory modalities activated distinct subfields within the cortical ribbon, separated by several millimeters. Columnar analysis was crucial in identifying each sensory-specific subfield, facilitating targeted laminar analysis within each distinct area to ensure that the unique laminar profiles of each subfield were not conflated (Fig. 2).

## Speeding up layer fMRI acquisition

With advancements in sequence methods and analysis strategies as reviewed previously, laminar fMRI has been successfully implemented at 7 T for human brain research, although it involves significant tradeoffs such as prolonged TR and consequential low temporal resolution. Most laminar fMRI research currently achieves resolutions of ~0.7–0.8 mm, enabling the broad investigation of three functional layers: superficial depths encompassing supragranular layers (I to III), middle depths representing the granular layer (IV), and deep depths including infragranular layers (V and VI) (Waehnert *et al*., 2014).

Advancing layer-fMRI to half-millimeter or higher spatial resolutions, while also aiming for high temporal resolution with whole-brain coverage, introduces substantial challenges. Higher spatial resolution leads to larger matrix sizes, while acquisition length is constrained by T_2_* decay time, resulting in extended TRs and low temporal resolution. For example, achieving half-millimeter resolution fMRI is possible with segmented EPI (Stirnberg and Stöcker, 2020), but at the cost of significantly longer acquisition time. To push spatial and temporal resolutions further while expanding brain coverage, overcoming existing limits on acceleration—how many *k*-space lines need to be sampled or how much we can undersample *k*-space—and readout speeds—how fast each *k*-space readout line can be collected—is essential, as extending the TR beyond practical use is otherwise unavoidable.

**Overcoming current acceleration limit**

To enhance the time efficiency of laminar fMRI acquisitions, advanced high-speed acceleration techniques are crucial. Deep-learning (DL) reconstruction techniques, particularly those that are self-supervised, have shown promise for enabling up to 20-fold acceleration while maintaining signal fidelity comparable to standard 10-fold accelerated fMRI acquisitions (Demirel *et al*., 2021; Gulle *et al*., 2023). In addition to apply acceleration only in spatial *k*-space, another promising approach is to apply acceleration and reconstruction in spatiotemporal (*k,t*)-space, using a low-rank approach (Chen *et al*., 2023; Chiew *et al*., 2015). It leverages the temporal information during image reconstruction and can provide a greater degree of acceleration than time-independent methods. In future, it is crucial to test how these high accelerations affect laminar specificity, ensuring that the ultra-high-resolution laminar signature remains preserved in data reconstructed from highly undersampled *k*–*t* space.

**Fast readout speed**

The readout speed is constrained by the gradient system and the threshold of peripheral nerve stimulation (PNS), which are the hardware and physiological constraints. Using current 7 T systems with body gradients, given the typical size of a human head and the constraints set by PNS, the echo spacing of 0.8 mm resolution EPI readout is around 1.0–1.1 ms. By contrast, this echo spacing could be halved with the advent of next-generation human brain 7 T scanner equipped with head-only gradient coils (Feinberg *et al*., 2023). This kind of scanner, designed specifically for human brain research, confine the gradient coil's influence to the head, allowing faster gradient switching and reducing PNS risks, thus enhancing the performance for EPI, which is crucial in fMRI. Such advancements facilitate faster EPI readouts, minimize T_2_* decay and geometric distortions, and enable higher spatial resolution and improved SNR. With the integration of the head-only gradient system, noninvasive human brain imaging can better achieve half-millimeter or even sub-half-millimeter resolutions (Feinberg *et al*., 2023). This unprecedented spatial resolution in human fMRI is instrumental in identifying functional activity across histologically distinct cortical layers, mapping columnar organization, and detailing vascular and subcortical structures for fMRI analysis, thereby enhancing our understanding of brain function at this micro- to mesoscale resolution.

## Conclusions

This article provides an overview of the current state and future prospects of laminar fMRI, focusing on technical challenges and innovative solutions shaping the field. We listed the methodological challenges identified in laminar fMRI, and in response, reviewed typical fMRI contrast methods utilized in laminar fMRI measurement, emphasized the importance of acquisition-matched anatomical imaging, introduced analysis strategies to improve sensitivity and specificity, and, last, provided technical perspectives of layer fMRI acquisitions aiming at higher spatiotemporal resolution. As we navigate through these laminar fMRI methodologies, the field remains dynamic and evolves rapidly. It is still not apparent which contrast methods, analysis pipelines, and acquisition designs will ultimately prevail and come into common practice for future laminar fMRI research. Nevertheless, the relentless progression in MRI technology is gradually overcoming current limitations, advancing us toward precise *in vivo* imaging of human brain function, extending from macroscopic to mesoscopic scales and potentially to the microscopic level in the foreseeable future.
